# A roadmap towards implementing health technology assessment in Oman

**DOI:** 10.1108/JHOM-01-2024-0012

**Published:** 2024-07-12

**Authors:** Ibrahim Al Rashdi, Sara Al Balushi, Alia Al Shuaili, Said Al Rashdi, Nadiya Ibrahim Al Bulushi, Asiya Ibrahim Al Kindi, Qasem Al Salmi, Hilal Al Sabti, Nada Korra, Sherif Abaza, Ahmad Nader Fasseeh, Zoltán Kaló

**Affiliations:** Directorate General of Medical Supplies, Ministry of Health Oman E-Library, Muscat, Oman; Director General of Planning and Studies, Ministry of Health Oman E-Library, Muscat, Oman; Senior Consultant Cardiothoracic Surgeon, Sultan Qaboos University Hospital, Muscat, Oman; Syreon Middle East, Alexandria, Egypt; Faculty of Pharmacy, Alexandria University, Alexandria, Egypt; Syreon Research Institute, Budapest, Hungary; Center for Health Technology Assessment, Semmelweis University, Budapest, Hungary; Minister of Health Office, Ministry of Health Oman E-Library, Muscat, Oman

**Keywords:** Health technology assessment, Oman, HTA, Evidence-based health policy, Healthcare system

## Abstract

**Purpose:**

Health technologies are advancing rapidly and becoming more expensive, posing a challenge for financing healthcare systems. Health technology assessment (HTA) improves the efficiency of resource allocation by facilitating evidence-informed decisions on the value of health technologies. Our study aims to create a customized HTA roadmap for Oman based on a gap analysis between the current and future status of HTA implementation.

**Design/methodology/approach:**

We surveyed participants of an advanced HTA training program to assess the current state of HTA implementation in Oman and explore long-term goals. A list of draft recommendations was developed in areas with room for improvement. The list was then validated for its feasibility in a round table discussion with senior health policy experts to conclude on specific actions for HTA implementation.

**Findings:**

Survey results aligned well with expert discussions. The round table discussion concluded with a phasic action plan for HTA implementation. In the short term (1–2 years), efforts will focus on building capacity through training programs. For medium-term actions (3–5 years), plans include expanding the HTA unit and introducing multiple cost-effectiveness thresholds while from 6–10 years, publishing of HTA recommendations, critical appraisal reports, and timelines is recommended.

**Originality/value:**

Although the HTA system in Oman is still in its early stages, strong initiatives are being taken for its advancement. This structured approach ensures a comprehensive integration of HTA into the healthcare system, enhancing decision-making and promoting a sustainable, evidence-based system addressing the population’s needs.

## Introduction

Since the establishment of the Ministry of Health (MoH) in 1970, Oman has undergone a significant transformation in its healthcare sector aiming at providing free universal healthcare to the population (Al Dhawi *et al.*, 2007; Alshishtawy, 2010). This development has significantly improved health outcomes (Alshishtawy, 2010). Infant mortality in Oman decreased from 118 per 1,000 live births before 1970 to 16 in 2002, and further to 8.8 by 2022. Concurrently, life expectancy reached up to 77 years by 2023 in the Omanis population (Al Dhawi *et al.*, 2007; Ministry of Health, 2022). Additionally, it has promoted uniform access to healthcare services throughout the country (World Health Organization, 2008).

The health system is primarily government-funded, with limited cost-sharing for non-Oman citizens through employer mandates (Alshishtawy, 2010). The MoH remains the main provider, offering universal coverage to all Oman and non-Oman citizens working in the public sector (Alshishtawy, 2010).

Oman has a national reference for economic and social planning for the period 2021–2040 known as “Oman Vision 2040” (Food and Agriculture Organization of the United Nations, 2021). One of its goals is to establish a transparent healthcare system that promotes justice and delivers high-quality services (Food and Agriculture Organization of the United Nations, 2021). Furthermore, it aims to maintain sustainable and continuous health funding and to foster leadership in scientific research and health innovation through capacity building (Food and Agriculture Organization of the United Nations, 2021).

In 2020, Oman spent around 5.3% as a percentage of its gross domestic product (GDP) on healthcare (World Bank, 2023). In response to the desired goals of the 2040 Oman Vision (Food and Agriculture Organization of the United Nations, 2021), it is anticipated that healthcare expenditure will increase in the upcoming years. However, achieving such goals requires not only the allocation of additional financial resources but also the efficient utilization of existing resources (Sun *et al.*, 2017).

Although Oman is considered a high-income country, many factors can have a negative impact on the sustainability of its healthcare system (Al Dhawi *et al.*, 2007). The increased cost of new medicines is one thing, particularly related to technologies with high upfront costs, such as genetic therapies or new-generation vaccines (Al Dhawi *et al.*, 2007; Carr and Bradshaw, 2016). Furthermore, the accelerated pace of innovation results in an increasing number of new technologies, which cannot be made accessible to all potentially eligible even in the most affluent countries (Grutters *et al.*, 2019; Chapman *et al.*, 2020). As Oman’s health system provides universal coverage, increased customer expectations can further add to the complexity of maintaining the sustainability of healthcare financing (Al Dhawi *et al.*, 2007).

Therefore, public payers – those responsible for paying for the reimbursement of health technologies out of the governmental budget – should seek to maximize health benefits from the available resources which can be supported by health technology assessment (HTA) implementation. HTA is a multidisciplinary process that uses scientific evidence and proven methodologies in an explicit approach to determine the value of health technology at different points in its lifecycle (O’Rourke *et al.*, 2020). Therefore, HTA provides the evidence needed to guide the reimbursement of new health technologies (Joore *et al.*, 2020).

The benefits of HTA implementation outweigh its potential drawbacks, as its primary objective is the efficient allocation of resources and not cost-saving (Mueller *et al.*, 2017). HTA implementation facilitates the establishment of a transparent and responsive decision support system (Fasseeh *et al.*, 2022b). By balancing equity, quality in healthcare, and decision-making efficiency, HTA implementation enhances health gain and financial protection for individuals (Fasseeh *et al.*, 2022b; Mueller *et al.*, 2017). To implement recommendations proposed by experts and monitor for any potential modification, a formal and institutionalized system of HTA is necessary (Mueller *et al.*, 2017).

To establish formal HTA in Oman, a clearly designed roadmap that fits the local settings should be established. The aim of our study is to provide a tailor-made HTA implementation roadmap in Oman, through exploring the gaps between the current and preferred future HTA environment. The gap analysis provides the basis for an action plan for HTA implementation roadmap.

## Methods

We used a scorecard survey covering eight HTA domains (Kaló *et al.*, 2016). The survey was disseminated to participants of an advanced HTA training program from various public and private health institutions including the Ministry of Health in Muscat, Sultan Qaboos Comprehensive Cancer Care and Research Centre (SQCCCRC) healthcare institutions in Seeb, and the Royal Hospital in Muscat. Based on the survey results, draft recommendations were developed for each HTA domain. Next, the draft recommendations were validated and modified by high-level decision-makers during a round table discussion. The discussion also determined the feasible timeline for implementing the recommendations in three phases: short-term, mid-term, and long-term.

A Bing-based artificial intelligence tool was used for the editing of limited sentences in the manuscript, but not utilized in the creation, development, or generation of the manuscript (Microsoft Bing, n.d.).

### Survey

#### Study design and setting

A scorecard designed to support the formulation of HTA roadmaps in individual countries was adopted in Oman. Several countries have already explored the gap between the current and preferred future status of HTA implementation using the same scorecard including Egypt, Jordan, Romania, Turkey and Ukraine (Csanádi *et al.*, 2019; Atikeler *et al.*, 2023; Rais *et al.*, 2020; Fasseeh *et al.*, 2022a; Almomani *et al.*, 2021). Additionally, the survey was conducted in Latin America and in the Middle East and North Africa region (Rosselli *et al.*, 2017; Fasseeh *et al.*, 2020). In our study, the same survey template was used to allow comparability across different countries.

The survey assessed the current and preferred future status of HTA implementation in 8 domains: capacity building; HTA funding; HTA legislation; scope of HTA; decision criteria; quality and transparency; use of local data; and international collaboration.

The capacity-building domain explores how to increase the number of trained HTA experts through human capacity-building initiatives. The funding domain evaluates the necessary financial support to conduct HTA research or to critically appraise the submitted HTA dossiers. The HTA legislation domain addresses the role of HTA in the decision-making process and the necessary institutional structure. The scope of HTA implementation domain asks about the types of health technologies to be assessed. The decision criteria domain focuses on which criteria to be included in the HTA process to drive policy decisions (e.g. cost-effectiveness or budget impact analysis). The remaining domains address the necessity of local data, the quality assurance and transparency of HTA documents, and international collaboration in producing HTA documents and implementing HTA training.

#### Study population and sampling

The survey was disseminated on the 27th of October 2022 in Muscat during an advanced capacity-building program for HTA. The terms in the survey were discussed with participants, then the survey was disseminated electronically through a proprietary platform. The selection of participants for this study was conducted through convenience sampling, ensuring the inclusion of individuals from a diverse range of institutions within Oman who possess knowledge of HTA. These individuals were nominated by their institution’s head, guaranteeing their familiarity with HTA. A target sample size of 30 survey respondents (with a bare minimum of 20 respondents) was proposed based on the adaptation of the same survey methodology in other countries (Rais *et al.*, 2020; Csanádi *et al.*, 2019).

#### Data collection and statistical analysis

Survey responses were aggregated using simple descriptive statistics and presented as percentages.

Aggregate survey responses were analyzed to produce a list of draft recommendations. These draft recommendations, formulated by the research team, were based solely on survey responses that received endorsement from over half of the participants and are presented in Table 3. These recommendations were subsequently presented to high-level decision-makers during a roundtable discussion for further modification and validation. The primary aim of these draft recommendations was to bridge the existing gap and move towards the preferred state of HTA implementation, as highlighted across the eight surveyed domains.

#### Ethical considerations including consent

Survey responses were used anonymously in publications based on participants’ consent. Respondents provided their consent by answering the first question in the survey. All responses were valid.

### Round table discussion

To validate and modify the draft recommendations, a round table discussion was conducted on the 5th of March 2023. Experts involved in the discussion were affiliated to the Ministry of Health and Sultan Qaboos Comprehensive Cancer Care and Research Centre (SQCCCRC) healthcare institutions, and the Royal Hospital.

The discussion began with the international moderator, who had designed the survey template and coordinated similar research projects in various countries, introducing the research objective and presenting the survey results and the initial recommendations to high-level decision-makers. Next, high-level decision-makers evaluated the initial recommendations for their feasibility from different perspectives (technical, legal, cultural, and political). Furthermore, decision makers established the best chronological order of recommended actions by breaking them down into three distinct phases, short-term (1–2 years), mid-term (3–5 years), and long-term (6–10 years).

## Results

### Survey results and validation

#### Demographics of survey respondents

A total of 21 responses were collected. Twenty (95%, *n* = 20) participants were employed in the public sector and only 1 (5%, *n* = 1) was employed in the private sector. The majority of survey respondents (81%, *n* = 17) had their primary education in pharmaceutical sciences. Details of the survey respondents are presented in Table 1.

The following paragraphs describe the results for each domain narratively, with Table 2 presenting the numerical data per domain. The list of draft recommendations based on survey responses is presented in Table 3 while Table 4 outlines recommended actions in each domain to reach the preferred HTA status in 10 years in Oman.

#### Capacity building

*Survey results.* One of the critical elements in implementing HTA is capacity building reflected in the presence of highly skilled professionals in a multidisciplinary team. According to the survey results, most respondents (76%, *n* = 16) reported that capacity building in Oman is currently limited to short courses only which are usually sponsored by pharmaceutical companies. The reliance on short courses may not be enough to induce hands-on training experience. All survey respondents preferred having permanent graduate and postgraduate programs on top of short courses in the future.

*Validation results (round table discussion).* The validation meeting confirmed the importance of HTA capacity building in Oman. In the short term, train-the-trainers programs were recommended where Omanis are sent abroad to apply for postgraduate programs in HTA and health economics. Furthermore, it was recommended not to restrict training for only HTA doers, but to include short courses for other stakeholders involved in the decision process as healthcare professionals. Short courses can also be included in their residency program. HTA public awareness was encouraged by experts to support transparency, allowing the public to understand on what basis the decision was made. Public awareness was suggested to be implemented through YouTubers who can discuss scientific materials in a simple way to reach public understanding. In the long term – depending on the progress achieved in HTA and Oman’s small population – the decision will be made whether there is a need to develop local master’s degrees and doctorate degrees in Oman or just diplomas and short courses are enough to support capacity building.

#### HTA funding

*Survey results.* For HTA implementation sustainable funding is a must. Funding is required to assess health technologies by conducting cost-effectiveness analysis and budget impact analysis. Additionally, funding is also required for the critical appraisal of already assessed health technologies to validate the results of the conducted cost-effectiveness studies and develop policy recommendations based on the conclusion reached. Funding can be supplied through public, private or a mix of both organizations.

Survey results indicated limited current funding for the assessment and the critical appraisal of health technologies as reported by more than 80% (*n* = 17) of respondents. In the future, regarding the critical appraisal of health technologies, the preference of survey respondents was split between having dominant public funding (52%, *n* = 11) and private funding through submission fees (43%, *n* = 9). For the assessment of health technologies in the future, most survey respondents (95%, *n* = 20) preferred dominant or at least sufficient public funding.

*Validation results (round table discussion).* Since Oman will adopt a single HTA system (as explained in the “HTA legislation” section below), experts recommended that the critical appraisal should be dominantly funded by the Ministry of Health (government) with contributions from the private sector through submission fees. On the other hand, the manufacturer will be responsible for the assessment of health technologies. However, in the long term, high public health priority technologies which are not of interest to the manufacturers (e.g. awareness campaigns, pharmaceutical dose reduction initiatives) will be assessed by the government.

#### HTA legislation

*Survey results.* Regarding the role of HTA in the decision-making process, nearly half (43%, *n* = 9) of the respondents indicated that international HTA evidence is considered in decision-making while the other half (52%, *n* = 11) reported the absence of a formal role of HTA. In the future, almost all (95%, *n* = 20) respondents preferred relying on local HTA evidence instead of international evidence or even mandating its use in policy decisions.

For the organizational structure, more than half (67%, *n* = 14) of the respondents indicated that currently, there is no public committee or institute responsible for the appraisal process, while only 29% (*n* = 6) reported the presence of a committee appointed for the appraisal process either with or without academic support. In the future, the majority of respondents (81%, *n* = 17) preferred the presence of a national HTA institute with academic support (38%, *n* = 8) or several HTA bodies with central coordination (48%, *n* = 9).

*Validation results (round table discussion).* Experts recommended the establishment of a national HTA unit under the umbrella of the Ministry of Health within the first two years. This unit will further expand with the increasing scope of assessed health technologies. As for critical appraisals, the Ministry of Health will take the lead in appraising HTA dossiers in the first 2 years. While from 3–5 years, if the capacity of appraisals increases, outsourcing with a third party might be required.

#### Scope of HTA implementation

*Survey results.* This domain discusses the types of health technologies to be assessed by the HTA body and the depth of assessment. Based on the survey results, more than half (57%, *n* = 12) of the respondents reported that health technologies are not assessed by HTA bodies in Oman and 33% (*n* = 7) reported that only new technologies with high budget impact are assessed. In the future, almost all respondents (95%, *n* = 20) would prefer to perform HTA for all new technologies and to revise previous pricing and reimbursement decisions.

Almost half of the survey respondents (48%, *n* = 10) indicated that currently only pharmaceuticals are assessed by HTA bodies (if HTA is applied). In the future, most respondents preferred expanding the scope of HTA to different technologies, including pharmaceuticals (81%, *n* = 17), medical devices (86%, *n* = 18), prevention programs (90%, *n* = 19), and surgical interventions (81%, *n* = 17).

*Validation results (round table discussion).* Experts recommended following a stepwise approach in expanding the scope of HTA implementation. From 1–2 years, the focus will be on only new pharmaceuticals with a high budget impact. If human capacity allows for assessing more technologies, then medical devices can be assessed as well in the first 2 years. From 3–5 years, the scope of technologies will expand to primarily include medical devices and surgical interventions. While, in the long term (6–10 years), prevention programs will be further added, together with revising previous policy decisions. Revising previous policy decisions was deferred to a later stage (6–10 years) because the assessments conducted using HTA in the 5 years before this time will facilitate and simplify the revision process as decisions might not change significantly upon revision.

#### Decision criteria

*Survey results.* The decision-making process considers several criteria that aid in reaching a decision. Currently, in Oman, cost-effectiveness and budget impact analysis were the most common criteria reported to be considered in the policy process by 38% (*n* = 8) and 33% (*n* = 7) of respondents, respectively.

In the future, respondents preferred considering more criteria in the decision-making process including unmet medical need (52%, *n* = 11), health care priority (57%, *n* = 12), therapeutic value (71%, *n* = 15), on top of cost-effectiveness (81%, *n* = 17), and budget impact (81%, *n* = 17). Respondents indicated that currently explicit multi-criteria decision analysis (MCDA) framework is not applied. However, in the future 95% (*n* = 20) would prefer applying MCDA to judge the value of selected health technologies based on multiple criteria.

For the cost-effectiveness threshold, most survey respondents (81%, *n* = 17) indicated the absence of a cost-effectiveness threshold in Oman. In the future, 95% (*n* = 20) of survey respondents would prefer adopting cost-effectiveness thresholds, of which 81% (*n* = 17) preferred explicit thresholds over implicit ones. Out of the 81% (*n* = 17) preferring explicit thresholds, 57% (*n* = 12) would like to adopt explicit soft thresholds.

Explicit hard thresholds rely only on the cost-effectiveness of a health technology for resource allocation decisions, while explicit soft thresholds are more flexible allowing negotiations and other criteria to be considered other than cost-effectiveness (Eichler *et al.*, 2004). In other words, if the incremental cost-effectiveness ratio (ICER) of a technology exceeds the hard threshold value, negotiation is not permitted, and the technology is not reimbursed. However, with soft thresholds, there is a chance for the reimbursement of technologies that are not cost-effective after price negotiation or taking into account other criteria (Eichler *et al.*, 2004).

*Validation results (round table discussion).* Experts were aligned on the use of explicit soft thresholds and MCDA. As GDP per capita is the most frequently applied reference point for establishing cost-effectiveness thresholds (Griffiths *et al.*, 2015), they recommended the use of multiple thresholds based on 1–3 times the GDP per capita as a starting point in the first 2 years. During this period, efforts will be made to establish a cost-effectiveness threshold specific to Oman by the third year (3–5 years). Regarding MCDA, it was recommended to implement MCDA for selected decision problems as a pilot in the first 2 years and expand its use in 3–5 years.

#### Quality and transparency of HTA implementation

*Survey results.* To improve the quality of HTA, several approaches are available, including publishing methodological guidelines for the assessment process, publishing appraisal reports together with a critical appraisal checklist and following up on previous recommendations. In Oman, the great majority of respondents (86%, *n* = 18) reported that none of the quality elements are utilized to improve HTA quality. In the future, they preferred having a publicly available critical appraisal checklist (76%, *n* = 16), published methodological guidelines (52%, *n* = 11) and following up on HTA recommendations (33%, *n* = 7).

Technology assessment, critical appraisal reports, and HTA recommendations are currently not accessible to the public as reported by 95% (*n* = 20) of the respondents. However, 90% (*n* = 19) of respondents preferred them to be published in the future. When it came to the timelines of HTA submissions and issuing recommendations, 95% (*n* = 19) of the respondents reported the absence of transparent timelines. However, in the future 86% (*n* = 18) of respondents preferred having transparent timelines for HTA submissions as well as issuing recommendations.

*Validation results (round table discussion).* Experts recommended pilot testing during the first year of HTA implementation. Pilot testing, involving testing the HTA under real conditions and collecting feedback, will allow experts to decide on clear timelines starting from the second year. As for transparency, experts recommended to start by publishing recommendations of the appraisal process during the first 5 years followed by publishing full appraisal reports from 6–10 years.

#### Local data

*Survey results.* Local data was not required in the current HTA process as reported by 74% (*n* = 14) of survey respondents. In the future, most survey respondents (81%, *n* = 17) preferred mandating the use of local data for HTA evidence together with assessing the transferability of international data in the absence of local data. More than 70% (*n* = 15) of survey respondents reported limited availability or accessibility to local real-world data. In the future, 95% (*n* = 20) of respondents preferred allocating more resources to establishing patient registries in certain disease areas and allowing payer’s databases to be accessible for HTA doers.

*Validation results (round table discussion).* For the short term, experts recommended using the currently available local data in addition to building a data warehouse to support HTA which can be ready to use from 3–5 years. Furthermore, the currently available electronic claims database in Oman can serve as a source for local cost and resource utilization data after some modification in its structure limiting the dependence on international data and assessing their transferability.

#### International collaboration

*Survey results.* All survey respondents reported that currently they are not involved in any joint international work. In the future, 90% (*n* = 18) of respondents preferred the adaptation of HTA materials developed by international HTA bodies. Currently, all respondents indicated limited participation in international courses related to HTA while in the future, 86% (*n* = 18) of respondents preferred participating or even developing international HTA courses.

*Validation results (round table discussion).* Experts recommended collaboration through the Gulf Cooperation Council (GCC) and the Gulf Health Council. Oman can therefore benefit from exchanging experiences and reusing HTA materials developed by other GCC countries. Experts also recommended collaboration in HTA training through the participation or hosting of international training.

## Discussion

As with other Middle East and North African countries (MENA) (Fasseeh *et al.*, 2020), initiatives have been taken to implement HTA in Oman. Due to the strong political commitment, workshops and webinars led by external experts were conducted to introduce HTA in Oman (Oman Observer, 2019, 2021). These initiatives highlighted the need for an HTA roadmap to support evidence-informed decision-making. Consequently, a roadmap was developed based on experts’ survey and stakeholder recommendations.

The survey results came from a scorecard that was previously used in many countries (Csanádi *et al.*, 2019; Atikeler *et al.*, 2023; Rais *et al.*, 2020; Fasseeh *et al.*, 2022a; Almomani *et al.*, 2021) to support HTA implementation. Using the same scorecard allows comparisons among different countries, which showed several commonalities in some key elements of HTA implementation but also some diversity in other aspects.

With regard to capacity building, experts in Oman recommended providing regular short courses and train-the-trainers programs in the short-term (1–2 years), which was consistent with the recommendations of other countries like Jordan (Almomani *et al.*, 2021). However, Omani experts have placed a greater emphasis on increasing public awareness of HTA, reflecting a focus on transparency that aligns with Oman’s Vision 2040. While many countries that applied the scorecard agreed to develop master’s and doctorate degree programs in the long term (6–10 years) (Almomani *et al.*, 2021; Fasseeh *et al.*, 2022a), Omani experts expressed hesitation about developing such programs due to the country’s small population size, which may not support the sustainability of the programs. Consequently, the decision to develop these programs has been deferred for at least 5 years.

In terms of funding, Omani experts expressed a preference for adopting a single HTA system in which the critical appraisal of health technologies will be conducted and funded by the Ministry of Health, while assessments will be performed by the manufacturers. This choice reflects the political will to implement HTA, which was reflected in the recommendations of other countries that used the same scorecard like Ukraine (Csanádi *et al.*, 2019). However, what sets Oman apart, is that experts have agreed that, in the midterm, there may be outsourcing with third parties to conduct appraisals due to uncertainty about the availability of sufficient human resources.

Considering that most Middle Eastern countries have fragmented healthcare systems, (Fasseeh *et al.*, 2020, 2022a; Almomani *et al.*, 2021) most countries that used the same scorecard expressed a preference for the presence of multiple HTA bodies.(Fasseeh *et al.*, 2020, 2022a; Almomani *et al.*, 2021). Even when survey results indicated a preference for a central system, experts typically agreed during the feasibility and validation phase to establish multiple HTA bodies to avoid political conflicts that may arise (Fasseeh *et al.*, 2022a; Almomani *et al.*, 2021). However, in Oman, decision-makers from different institutions agreed to establish a central HTA body under the umbrella of the Ministry of Health, rather than having multiple agencies.

Regarding the scope of HTA implementation, the decision to initially focus on innovative pharmaceuticals with high budget impact was consistent with the results observed in Egypt and Jordan, (Fasseeh *et al.*, 2022a; Almomani *et al.*, 2021) where the scope expands to also include the revision of previous reimbursement decisions in the future.

For cost-effectiveness threshold (CET), Omani experts aim to establish a validated cost-effectiveness threshold within a timeframe of three to five years. Therefore, work must commence promptly to allow for the completion of a pilot phase and subsequent adoption within the specified timeframe.

Omani experts decided to publish appraisal reports within 10 years, in addition to previously published appraisal recommendations and timelines. The decision to postpone publishing critical appraisal reports to long-term (6–10 years) was made to allow more time for improving their quality. In contrast, Egypt recommended to publish methodological guidelines and timelines as a midterm plan (3–5 years) (Fasseeh *et al.*, 2022a).

In terms of local data, experts indicated that there is an existing claims database in Oman. This represents a strong starting point for acquiring reliable local data. However, some modifications to its structure will be necessary to meet the needs of HTA. Once modified, the reliance will be mainly on local data. Finally, regarding international collaboration, experts recommended participation in, or even hosting of, international training, as well as the exchange of information through the Gulf Cooperation Council (GCC).

National healthcare systems, including Oman’s, do not have sufficient resources to fund all new and old health technologies. Therefore, they must choose among different health technologies. To choose effectively, HTA compares health technologies based on their costs, clinical effectiveness, side effects, and other factors (Joore *et al.*, 2020). HTA then provides evidence to support and guide decision-makers on the reimbursement of health technologies.

Oman’s 2040 vision has established ambitious healthcare system objectives (Food and Agriculture Organization of the United Nations, 2021). Such objectives include the creation of a transparent system, providing high-quality services, and achieving universal health coverage (Food and Agriculture Organization of the United Nations, 2021). However, with challenges facing healthcare systems, including expensive treatments, the rapid pace of innovation, the emergence of genetic therapies, and the growing number of orphan drugs, it is of great importance that Oman takes proactive measures to ensure the efficient allocation of resources and the adoption of new cost-effective health technologies through HTA.

Although there are several HTA implementation frameworks in the scientific literature describing HTA roles and implementation plans, HTA roadmaps cannot be fully transferred from other countries due to various factors, such as the size of the country, GDP per capita, the health system structure, legal, cultural, and political aspects (Kaló *et al.*, 2016). Therefore, for Oman to implement HTA, it needs to have its own roadmap based on its health policy objectives.

Our HTA roadmap outlines a strategy with specific timelines for the adoption of HTA (Table 4). Moreover, creating a roadmap is only a step in HTA implementation. We recommend regular monitoring of actions to allow for the revision of timelines or modification of certain action items. The HTA roadmap can also facilitate organized and streamlined implementation of HTA enabling progress to be monitored and facilitating a more rapid implementation process.

### Limitations

Our study had some limitations including the small sample size of survey respondents; however, we involved participants with rich knowledge about HTA and the number of survey participants was similar to studies which applied the same methodology in other countries. The low number of experts (sample size) implicated subgroup analysis by different employment statuses (public vs private), where the private sector was underrepresented in the study. Furthermore, patient representatives were not included due to the under-established patient organizations currently available in Oman. Finally, we acknowledge that the majority of our sample had their primary education in pharmaceutical sciences, with minimal representation from other sectors. This is because experts in HTA and health economics often emerge from such disciplines.

## Conclusions

The phasic HTA implementation plan for Oman identifies a strategic progression through immediate and long-term goals over a 10-year period. In the short term (1–2 years), the focus is on building capacity through training programs and integrating HTA education into healthcare professional curriculums. Additionally, early financing efforts are crucial for the assessment (especially of pharmaceuticals with high budget impact) and the appraisal of health interventions.

For medium-term actions (3–5 years), the plan emphasizes increasing public awareness of HTA, broadening assessments to include medical devices and surgical interventions alongside pharmaceuticals, expanding the HTA unit to accommodate a larger scope of technologies, and developing a CET for Oman. Over a longer span (6–10 years), the strategy aims to further widen the HTA scope to cover an extensive range of healthcare technologies and ensure the publication of recommendations, critical appraisal reports, and clear timelines.

This structured approach ensures a comprehensive integration of HTA into the healthcare system, enhancing decision-making at various levels. Such foundational steps towards HTA integration in Oman have set the stage for an evolving healthcare system that optimizes health outcomes and resource allocation through evidence-informed decisions.

Moving forward, the action plan must undergo rigorous evaluation to identify areas of success and aspects requiring enhancement. This iterative process will ensure that HTA integration not only aligns with but also anticipates the dynamic needs of Oman’s healthcare landscape. By fostering an environment that encourages systematic evaluation, Oman can achieve a sustainable, evidence-based healthcare framework that effectively meets the needs of its population.

## Figures and Tables

**Table 1:** Demographics of survey respondents (*N* = 21)

	*N* (%)
*Main employment*	
Public sector	20 (95%)
Private sector	1 (5%)
*Field of work (public sector)*	
Decision-maker, policy-maker, the public payer (Social Security Institution), Ministry of Health (potential HTA user)	7 (33%)
Public health care provider (e.g. clinician)	13 (62%)
Other	1 (5%)
*Field of work (private sector)*	
Health care industry (e.g. pharmaceutical or medical device company)	1 (100%)
*Major training*	
Economics	3 (14%)
Pharmacy	17 (81%)
Other health care (e.g. nursing, dietetics)	1 (5%)
*Age*	
Below 30	2 (9.5%)
Between 30 and 50	19 (90.5%)

**Source(s):** Authors work

**Table 2:** Aggregated results of valid responses from HTA implementation survey (scorecard)

Consent	Yes	No
	*n* (%)	*n* (%)
Hereby, I accept that my anonymous answers can be aggregated and used in scientific presentations and publications	21 (100%)	0 (0%)

Question	Current status	Preferred status
	*n* (%)	*n* (%)
*1. HTA capacity-building*		
*a) Education*		
No training	5 (23.8%)	0 (0.0%)
Project-based training and short courses	16 (76.2%)	0 (0.0%)
Permanent graduate program with short courses	0 (0.0%)	7 (33.3%)
Permanent graduate and postgraduate program with short courses	0 (0.0%)	14 (66.7%)
*2. HTA funding*		
*a) Financing critical appraisal of technology assessment*		
No funding for critical appraisal of technology assessment reports or submissions	17 (85.0%)	1 (4.8%)
Dominantly private funding (e.g. submission fees) by manufacturers for the critical appraisal of technology assessment reports or submissions	3 (15.0%)	9 (42.9%)
Dominantly public funding for critical appraisal of technology assessment reports or submissions	0 (0.0%)	11 (52.4%)
*b) Financing health technology assessment (i.e. HTA research)*		
No public funding for technology assessment; private funding is not needed or expected	17 (81.0%)	1 (4.8%)
No or marginal public funding for research in HTA; private funding is expected	4 (19.0%)	0 (0.0%)
Sufficient public funding for research in HTA; private funding is also expected	0 (0.0%)	12 (57.1%)
HTA research is dominantly funded from public resources	0 (0.0%)	8 (38.1%)
*3. Legislation on HTA*		
*a) Legislation on the role of the HTA process and recommendations in the decision-making process*		
No formal role of HTA in decision-making	11 (52.4%)	1 (4.8%)
Dominantly international HTA evidence is taken into account in decision-making	9 (42.9%)	0 (0.0%)
International and additionally local HTA evidence is taken into account in decision-making	1 (4.8%)	10 (47.6%)
Local HTA evidence is mandatory in decision-making	0 (0.0%)	10 (47.6%)
*b) Legislation on organizational structure for HTA appraisal*		
There is no public committee or institute for the appraisal process	14 (66.7%)	1 (4.8%)
A committee is appointed for the appraisal process	5 (23.8%)	0 (0.0%)
The committee is appointed for the appraisal process with the support of academic centers and independent expert groups	1 (4.8%)	2 (9.5%)
A public HTA institute or agency is established to conduct a formal appraisal of HTA reports or submissions	1 (4.8%)	1 (4.8%)
Public HTA institute or agency is established to conduct a formal appraisal of HTA reports or submissions with the support of academic centers and independent expert groups	0 (0.0%)	8 (38.1%)
Several public HTA bodies are established without central coordination of their activities	0 (0.0%)	0 (0.0%)
Several public HTA bodies are established with central coordination of their activities	0 (0.0%)	9 (42.9%)
*4. Scope of HTA implementation*		
*a) Scope of technologies (multiple choice)*		
HTA is not applied to any health technologies	11 (52.4%)	1 (4.8%)
Pharmaceutical products	10 (47.6%)	17 (81.0%)
Medical devices	3 (14.3%)	18 (85.7%)
Prevention programs and technologies	2 (9.5%)	19 (90.5%)
Surgical interventions	1 (4.8%)	17 (81.0%)
Other scope of technologies	0 (0.0%)	3 (14.3%)
*b) Depth of HTA use in pricing and/or reimbursement decision of health technologies*		
HTA is not applied to any health technologies	12 (57.1%)	1 (4.8%)
Only new technologies with significant budget impact	7 (33.3%)	0 (0.0%)
Only new technologies	1 (4.8%)	0 (0.0%)
New technologies + revision of previous pricing and reimbursement decisions	1 (4.8%)	20 (95.2%)
*5. Decision criteria*		
*a) Decision categories (multiple choice)*		
None of the below categories are applied	6 (28.6%)	0 (0.0%)
Unmet medical need	4 (19.0%)	11 (52.4%)
Healthcare priority	4 (19.0%)	12 (57.1%)
Assessment of therapeutic value	5 (23.8%)	15 (71.4%)
Cost-effectiveness	8 (38.1%)	17 (81.0%)
Budget impact	7 (33.3%)	17 (81.0%)
Other decision categories	0 (0.0%)	1 (4.8%)
*b) Decision thresholds*		
Thresholds are not applied	17 (81.0%)	1 (4.8%)
Implicit thresholds are preferred	3 (14.3%)	3 (14.3%)
Explicit soft thresholds are applied in decisions	1 (4.8%)	12 (57.1%)
Explicit hard thresholds are applied in decisions	0 (0.0%)	5 (23.8%)
*c) Multi-criteria decision analysis*		
No explicit multi criteria decision framework is applied	20 (100.0%)	1 (4.8%)
Explicit multi criteria decision framework is applied	0 (0.0%)	20 (95.2%)
*6. Quality and transparency of HTA implementation*		
*a) Quality elements of HTA implementation (multiple choice)*		
None of the below quality elements are applied	18 (85.7%)	1 (4.8%)
Published methodological guidelines for HTA/economic evaluation	2 (9.5%)	11 (52.4%)
Regular follow-up research on HTA recommendations	1 (4.8%)	7 (33.3%)
A checklist to conduct a formal appraisal of HTA reports or submissions exists but not available for public	0 (0.0%)	9 (42.9%)
A published checklist is applied to conduct a formal appraisal of HTA reports or submissions	0 (0.0%)	16 (76.2%)
*b) Transparency of HTA in policy decisions*		
Technology assessment reports, critical appraisal and HTA recommendation are not published	20 (95.2%)	1 (4.8%)
HTA recommendation is published without details of technology assessment reports and critical appraisal	1 (4.8%)	1 (4.8%)
Transparent technology assessment reports, critical appraisals and HTA recommendations	0 (0.0%)	19 (90.5%)
*c) Timeliness*		
HTA submission and issuing recommendation have no transparent timelines	19 (95.0%)	0 (0.0%)
HTA submissions are accepted/conducted following a transparent calendar, but issuing recommendation has no transparent timelines	1 (5.0%)	3 (14.3%)
HTA submissions are accepted continuously and issuing recommendation has transparent timelines	0 (0.0%)	18 (85.7%)
*7. Use of local data*		
*a) Requirement of using local data in technology assessment*		
No mandate to use local data	14 (73.7%)	1 (4.8%)
The mandate of using local data in certain categories without the need for assessing the transferability of international evidence	4 (21.1%)	3 (14.3%)
The mandate of using local data in certain categories with the need for assessing the transferability of international evidence	1 (5.3%)	17 (81.0%)
*b) Access and availability of local data*		
Limited availability or accessibility to local real-world data	15 (71.4%)	0 (0.0%)
Up-to-date patient registries are available in certain disease areas, but payers’ databases are not accessible for HTA doers	4 (19.0%)	1 (4.8%)
Payers’ databases are accessible for HTA doers, patient registries are not available or accessible in the majority of disease areas	0 (0.0%)	3 (14.3%)
Up-to-date patient registries are available in certain disease areas and payers’ databases are accessible for HTA doers	2 (9.5%)	17 (81.0%)
*8. International collaboration*		
*a) international collaboration, joint work on HTA (joint assessment reports) and national/regional adaptation (reuse) (multiple choice)*		
No involvement in joint work; and no reuse of joint work or national/regional HTA documents from other countries	19 (100.0%)	1 (5.0%)
Active involvement in joint work (e.g. Eunet HTA Rapid REA, full Core HTA)	0 (0.0%)	5 (25.0%)
National/regional adaptation (reuse) of joint HTA documents	0 (0.0%)	7 (35.0%)
National/regional adaptation (reuse) of national/regional work performed by other HTA bodies in other countries	0 (0.0%)	18 (90.0%)
*b) International HTA courses for continuous education on HTA*		
Limited interest in (1) developing/implementing of and (2) participating at international HTA courses	18 (100.0%)	1 (4.8%)
Interest only in regular participation at international HTA courses	0 (0.0%)	2 (9.5%)
High interest in (1) developing/implementing of and (2) participating at international HTA courses	0 (0.0%)	18 (85.7%)

**Note(s):** For single choice questions, each expert chose 1 of the available options for the current status and 1 of the options for preferred status. *E.g. for question 1a: an expert chose “No training” in the current status and “Permanent graduate program with short courses” for the preferred status, this means he thinks there are currently no training programs, and he would prefer that in 10 years, there will be permanent graduate programs with short courses*

For multiple choice questions, each expert can choose more than one option for the current and preferred status. *E.g. for question 4a:* an expert chose “pharmaceuticals” and “medical devices” in the current status and “pharmaceuticals”, “medical devices”, “prevention programs” and “surgical interventions” for the preferred status. This means that currently he thinks that HTA is done for medical devices and pharmaceuticals and in the future, he prefers HTA to be done to surgical interventions and prevention programs as well

**Source(s):** Authors work

**Table 3:** Draft recommendations based on major gaps between the current and preferred status of HTA implementation in Oman according to the eight domains of the scorecard survey

Domain	Recommendations
Capacity building	More graduate and postgraduate HTA programs are recommended based on country-specific needs
HTA funding	Sufficient public funding should be allocated to HTA assessment. HTA critical appraisals should be funded partially but not only by submission fees (private funding)
Legislation on HTA	Establishment of a public HTA agency supported by academic efforts with major reliance on local HTA evidence. OR: Establishment of multiple HTA agencies with central coordination with major reliance on local HTA evidence
Scope of HTA implementation	Extending the scope of HTA from pharmaceuticals to non-pharmaceuticals is recommended in addition to revising previous policy decisions on top of evaluating new healthcare technologies
Decision criteria	For cost-effectiveness, explicit soft thresholds should be used. Cost-effectiveness, budget impact and therapeutic value should be the main decision categories. In addition, other criteria other should be considered by applying multi-criteria decision analysis (MCDA)
Quality and transparency	Using published methodological guidelines for HTA/economic evaluation and checklists for critical appraisal is recommended to improve HTA work quality. HTA reports, critical appraisals and HTA recommendations should be published to maintain transparency. In addition, HTA submission should be accepted continuously with clear timelines for recommendations
Use of local data	Local data should be mandatory and the assessment of transferability of international evidence when submitting the research for appraisal. Developing more patient registries and utilizing local claims data is recommended with the availability of an accessible electronic payer’s database
International collaboration	Organizing and participating in international HTA courses is highly recommended as well as adapting work performed by other HTA bodies

**Source(s):** Authors work

**Table 4:** Action plan for HTA implementation

	Action within 1–2 years	Action within 3–5 years	Actions from 6–10 years
Capacity building	• Train the trainers programs • Providing regular short courses for decision makers • Inclusion of short courses in the residency program of healthcare professionals	Increasing awareness of HTA among the public	Decide on the need for local academic programs (PhD or masters) implementation
HTA funding	• Public funding by the ministry of health will take the lead for the critical appraisal with minor submission fees from the private sector • Assessment should be financed mainly by pharmaceutical companies	Outsourcing with third parties might be needed with increased capacity for appraisal	Assessment of technologies with no interest to the manufacturer will be prioritized by the ministry of health and assessed according to importance
Legislation on HTA	Single national HTA unit under the umbrella of the MoH	Unit will expand with increasing scope of technologies	
Scope of HTA implementation	Start assessing innovative pharmaceuticals with high budget impact to support reimbursement decisions	Expand scope to medical devices and surgical interventions	Expand scope to prevention programs and revising HTA decisions
Decision criteria	Initially use 1–3x GDP per capita threshold value, apply multiple thresholds, and pilot MCDA in certain cases	Develop a CET for Oman, expand the use of MCDA	
Quality and transparency of HTA implementation	Recommendations only are published during the first 5 years	After 1 year of pilot testing, clear timelines will be established	Critical appraisal reports are published
Use of local data	Use available local data from electronic databases	Build local data warehouse	
International collaboration	Participation/hosting international training		Exchanging experience through Gulf Cooperation Council

**Source(s):** Authors work

## References

[ref001] Al Dhawi, A.A., West, D.J. Jr, Spinelli, R.J. and Gompf, T.A. (2007), “The challenge of sustaining health care in Oman”, The Health Care Manager, Vol. 26 No. 1, pp. 19-30, doi: 10.1097/00126450-200701000-00003. 17314623

[ref002] Almomani, E., Alabbadi, I., Fasseeh, A., Al-Qutob, R., Al-Sharu, E., Hayek, N., Tarawneh, M.R. and Kaló, Z. (2021), “Implementation road map of health technology assessment in middle-income countries: the case of Jordan”, Value in Health Regional Issues, Vol. 25, pp. 126-134, doi: 10.1016/j.vhri.2021.01.003. 34015521

[ref003] Alshishtawy, M.M. (2010), “Four decades of progress: evolution of the health system in Oman”, Sultan Qaboos University Medical Journal, Vol. 10 No. 1, pp. 12-22. 21509077 PMC3074664

[ref004] Atikeler, E.K., Fasseeh, A.N., Mantel-Teeuwisse, A.K., Çalışkan, Z., Öner, Z.G., Kızılay, H., Kalo, Z. and Goettsch, W. (2023), “Health technology assessment in Türkiye: current status and perspectives on future implementation”, Health Policy and Technology, Vol. 12 No. 1, 100701, doi: 10.1016/j.hlpt.2022.100701.

[ref005] Carr, D.R. and Bradshaw, S.E. (2016), “Gene therapies: the challenge of super-high-cost treatments and how to pay for them”, Regenerative Medicine, Vol. 11 No. 4, pp. 381-393, doi: 10.2217/rme-2016-0010. 27185544

[ref006] Chapman, S., Paris, V. and Lopert, R. (2020), “Challenges in access to oncology medicines: policies and practices across the OECD and the EU”, available at: https://www.oecd.org/health/health-systems/Addressing-Challenges-in-Access-to-Oncology-Medicines-Analytical-Report.pdf (accessed 4 April 2023)

[ref007] Csanádi, M., Inotai, A., Oleshchuk, O., Lebega, O., Alexandra, B., Piniazhko, O., Németh, B. and Kaló, Z. (2019), “Health technology assessment implementation in Ukraine: current status and future perspectives”, International Journal of Technology Assessment in Health Care, Vol. 35 No. 5, pp. 393-400, doi: 10.1017/s0266462319000679. 31583985

[ref008] Eichler, H.G., Kong, S.X., Gerth, W.C., Mavros, P. and Jönsson, B. (2004), “Use of cost‐effectiveness analysis in health‐care resource allocation decision‐making: how are cost‐effectiveness thresholds expected to emerge?”, Value in Health, Vol. 7 No. 5, pp. 518-528, doi: 10.1111/j.1524-4733.2004.75003.x. 15367247

[ref009] Fasseeh, A., Karam, R., Jameleddine, M., George, M., Kristensen, F.B., Al-Rabayah, A.A., Alsaggabi, A.H., El Rabbat, M., Alowayesh, M.S., Chamova, J., Ismail, A., Abaza, S. and Kaló, Z. (2020), “Implementation of health technology assessment in the Middle East and North Africa: comparison between the current and preferred status”, Frontiers in Pharmacology, Vol. 11, p. 15, doi: 10.3389/fphar.2020.00015. 32153393 PMC7046555

[ref010] Fasseeh, A.N., Elezbawy, B., Gamal, M., Seyam, A., Abourawash, A., George, M., Anwar, M., Amin, M., Khalifa, A.Y., Elshalakani, A., Hatem, A., Abdelhamid, S., Elsamouly, H., Fasseeh, N., Adel, R., Dawood, H., Abaza, S. and Kaló, Z. (2022a), “A roadmap toward implementing health technology assessment in Egypt”, Frontiers in Public Health, Vol. 10, 896175, doi: 10.3389/fpubh.2022.896175. 36582366 PMC9792961

[ref011] Fasseeh, A.N., Saragih, S.M., Hayek, N., Brodovska, S., Ismail, A., Elshalakani, A., Abaza, S., Obeng, G.D., Ameyaw, D. and Kalo, Z. (2022b), “Impact of health technology assessment implementation with a special focus on middle-income countries”, Health Policy and Technology, Vol. 11 No. 4, 100688, doi: 10.1016/j.hlpt.2022.100688.

[ref012] Food and Agriculture Organization of the United Nations (2021), “Oman vision 2040”, available at: https://www.fao.org/faolex/results/details/en/c/LEX-FAOC201987/#:∼:text=The%20Sultanate%20aims%20to%20(i,to%2010%20percent%20of%20GDP (accessed 12 May 2023).

[ref013] Griffiths, M., Maruszczak, M. and Kusel, J. (2015), “The who-choice cost-effectiveness threshold: a country-level analysis of changes over time”, Value in Health, Vol. 18 No. 3, A88, doi: 10.1016/j.jval.2015.03.517.

[ref014] Grutters, J.P., Govers, T., Nijboer, J., Tummers, M., Van Der Wilt, G.J. and Rovers, M.M. (2019), “Problems and promises of health technologies: the role of early health economic modeling”, International Journal of Health Policy and Management, Vol. 8 No. 10, pp. 575-582, doi: 10.15171/ijhpm.2019.36. 31657184 PMC6819627

[ref015] Joore, M., Grimm, S., Boonen, A., De Wit, M., Guillemin, F. and Fautrel, B. (2020), “Health technology assessment: a framework”, RMD Open, Vol. 6 No. 3, e001289, doi: 10.1136/rmdopen-2020-001289. 33148786 PMC7856136

[ref027] Kaló, Z., Gheorghe, A., Huic, M., Csanádi, M. and Kristensen, F.B. (2016), “HTA implementation roadmap in Central and Eastern European countries”, Health Economics, Vol. 25, pp. 179-192. 26763688 10.1002/hec.3298 PMC5066682

[ref016] Microsoft Bing (n.d.), available at: https://www.bing.com/chat (accessed 4 April 2023).

[ref017] Ministry of Health (2022), “Annual health report 2022: annual health report 2022”, available at: https://www.moh.gov.om/en/web/statistics/annual-reports/-/asset_publisher/aQdmeIpTn5pS/content/-2022?inheritRedirect=false&redirect=https%3A%2F%2Fwww.moh.gov.om%2Fen%2Fweb%2Fstatistics%2Fannual-reports%3Fp_p_id%3D101_INSTANCE_aQdmeIpTn5pS%26p_p_lifecycle%3D0%26p_p_state%3Dnormal%26p_p_mode%3Dview%26p_p_col_id%3Dcolumn-2%26p_p_col_count%3D1 (accessed 21 April 2024).

[ref018] Mueller, D., Tivey, D. and Croce, D. (2017), “Health-technology assessment: its role in strengthening health systems in developing countries”, Southern African Journal of Public Health, Vol. 2, pp. 6-11.

[ref019] Oman Observer (2019), “Health economics essential for Oman Vision 2040”, available at: https://www.omanobserver.om/article/6280/Local/health-economics-essential-for-oman-vision-2040 (accessed 13 May 2023).

[ref020] Oman Observer (2021), “Health technology assessment workshop begins”, available at: https://www.omanobserver.om/article/1119555/oman/health-technology-assessment-workshop-begins (accessed 13 May 2023).

[ref021] O’rourke, B., Oortwijn, W. and Schuller, T. (2020), “Announcing the new definition of health technology assessment”, Value in Health, Vol. 23 No. 6, pp. 824-825, doi: 10.1016/j.jval.2020.05.001. 32540240

[ref022] Rais, C., Kaló, Z., Csanádi, M. and Negulescu, V. (2020), “Current and future perspectives for the implementation of health technology assessment in Romania”, Health Policy and Technology, Vol. 9 No. 1, pp. 45-52, doi: 10.1016/j.hlpt.2019.11.007.

[ref023] Rosselli, D., Quirland-Lazo, C., Csanádi, M., De Castilla, E.M.R., González, N.C., Valdés, J., Abicalaffe, C., Garzón, W., Leon, G. and Kaló, Z. (2017), “HTA implementation in Latin American countries: comparison of current and preferred status”, Value in Health Regional Issues, Vol. 14, pp. 20-27, doi: 10.1016/j.vhri.2017.02.004. 29254537

[ref024] Sun, D., Ahn, H., Lievens, T. and Zeng, W. (2017), “Evaluation of the performance of national health systems in 2004-2011: an analysis of 173 countries”, PLoS One, Vol. 12 No. 3, e0173346, doi: 10.1371/journal.pone.0173346. 28282397 PMC5345793

[ref025] World Bank (2023), “Current health expenditure (% of GDP)”, available at: https://data.worldbank.org/indicator/SH.XPD.CHEX.GD.ZS?locations=OM (accessed 2 August 2023).

[ref026] World Health Organization (2008), “Demographic, social and health indicators for countries of the Eastern Mediterranean”, available at: https://apps.who.int/iris/handle/10665/116564 (accessed 4 April 2023).

